# Genome-wide association study of beef bull semen attributes

**DOI:** 10.1186/s12864-021-08256-z

**Published:** 2022-01-23

**Authors:** M. L. Butler, A. R. Hartman, J. M. Bormann, R. L. Weaber, D. M. Grieger, M. M. Rolf

**Affiliations:** Department of Animal Science Kansas State University, Manhattan, Kansas 66502 USA

## Abstract

**Background:**

Cattle production is dependent upon fertility because it results in producing offspring to offset production costs. A number of semen attributes are believed to affect fertility and are frequently measured as part of routine breeding soundness exams or semen collection procedures. The objective of this study was to perform a single-step genome-wide association study (ssGWAS) for beef bull semen attributes. Beef bull fertility phenotypes including volume (VOL), concentration (CONC), number of spermatozoa (NSP), initial motility (IMot), post-thaw motility (PTMot), three-hour post-thaw motility (3HRPTMot), percentage of normal spermatozoa (%NORM), primary abnormalities (PRIM), and secondary abnormalities (SEC) were obtained from two artificial insemination (AI) centers. A total of 1819 Angus bulls with 50,624 collection records were used for ssGWAS. A five-generation pedigree was obtained from the American Angus Association and consisted of 6521 sires and 17,136 dams. Genotypes on 1163 bulls were also obtained from the American Angus Association and utilized in ssGWAS.

**Results:**

A multi-trait animal model was used for the estimation of single nucleotide polymorphism (SNP) effects. Significant SNP were those with a -log_10_ *P*-value threshold greater than 4.0. Volume, CONC, NSP, IMot, PTMot, 3HRPTMot, %NORM, PRIM, and SEC have five, three, six, seven, two, six, six, and two genome-wide significant SNP, respectively.

**Conclusions:**

Several significant SNP were determined to be near or within quantitative trait loci (QTL) associated with beef bull semen attributes. In addition, genes associated with fertility were found to contain or be near the significant SNP found in the study. The results indicate there are regions of the genome that impact fertility, proving inclusion of genomic information into genetic evaluation should be advantageous for genetic improvement of male fertility traits.

## Background

Fertility is a complex trait which is affected by management [1–3], nutrition [4, 5], and genetics [6, 7]. The complexity of fertility may be one reason for the lack information available to beef seedstock producers for making fertility selection decisions. Other reasons may include that fertility data is not currently aggregated by beef breed associations and semen quality data from bull studs is largely proprietary. As genomic testing becomes more affordable and a part of regular management practices for seedstock producers, beef cattle producers become more willing and able to utilize the technology. Utilizing genomic technology in breeding decisions allows cattle producers to more confidently incorporate young, unproven sires into their breeding programs. While using young breeding stock increases the rate of genetic change, the risk of using these bulls is higher because their expected progeny differences (EPD) accuracies are lower. Beef cattle producers do not currently have a selection tool to confidently choose more fertile bulls, nor are current versions of SNP chips enriched in regions where important variants for male fertility exist because they are largely unstudied and unidentified.

However, thousands of semen records from beef bull semen collection facilities provide an opportunity to identify polymorphisms which may affect spermatogenesis, morphology, and motility of spermatozoa [8]. One way to truly increase the reproductive efficiency of beef cattle production is to build the capacity to identify young beef bulls with sperm abnormalities and low semen quality so that the bulls can be eliminated from the breeding population [8]. Dairy cattle research underscores the ability to capitalize on genomic technologies in these efforts, as researchers have identified multiple QTL regions [9–17] and candidate genes [18–24] associated with male and female fertility. Researchers have identified areas of the genome that are associated with fertility traits in beef bulls [25], but the number of studies is limited. Thus, the need for further validation is a necessity. The objective of this study was to perform a genome-wide association study for beef bull semen attributes and to identify quantitative trait loci (QTL) regions and genes likely associated with fertility traits in beef bulls.

## Methods

### Data collection

Phenotypic observations for 1819 Angus bulls were obtained from two bull semen collection facilities. The phenotypic observations for beef bull semen attributes included volume (VOL), concentration (CONC), number of spermatozoa (NSP), initial motility (IMot), post-thaw motility (PTMot), three-hour post-thaw motility (3HRPTMot), percentage of normal spermatozoa (%NORM), primary abnormalities (PRIM), and secondary abnormalities (SEC). Trait definitions are listed in Table 1. Data editing procedures were performed to ensure no VOL or CONC phenotypes were 0, all phenotypes recorded as a percentage ranged from 0 to 100, and all bulls had a registration number. After data editing procedures there were 50,624 total collection records utilized for the ssGWAS. Bull stud A contributed 48,131 collection records from 1570 bulls and bull stud B contributed an additional 2493 records from 256 bulls. Summary statistics are shown in Table 1.

**Table 1:** Trait list, definitions, and summary statistics of beef bull fertility traits collected at an artificial insemination. All post-thaw motility measures are observed the day after the sample is collected. All traits are measured within one hour except for three-hour post-thaw motility

Trait	Units	Definition	N	Mean	Std Dev	Minimum	Maximum
Volume (VOL)	mL	total amount of the ejaculate, measured by milliliters	44,431	7.96	4.22	0.10	74.00
Concentration (CONC)	Million/mL	relative amount of sperm cells per ejaculate, measured by a colorimeter	44,038	1021.16	495.02	10.00	3906.00
Number of spermatozoa (NSP)	Million	calculated by multiplying sperm concentration and semen volume; expressed in millions	44,418	49.18	16.16	0.00	100.00
Initial Motility (IMot)^a^	%	percentage of progressively swimming spermatozoa in the ejaculate immediately after collection	44,038	8004.18	5519.61	64.50	69,795.00
Post-thaw Motility (PTMot)^a^	%	percentage of progressively swimming spermatozoa in the ejaculate, measured within one hour of thawing	29,877	43.53	13.78	0.00	75.00
Three-hour post-thaw motility (3HRPTMot)^a^	%	percentage of progressively swimming spermatozoa in the ejaculate, measured three hours after thawing	8299	15.72	12.48	0.00	60.00
Percentage of Normal Spermatozoa (%NORM)^a^	%	percent morphologically normal spermatozoa	19,455	75.18	8.37	2.00	100.00
Primary Abnormalities (PRIM)^a^	%	percentage of spermatozoa with a defect to the head	19,452	13.00	7.53	0.00	100.00
Secondary Abnormalities (SEC)^a^	%	percentage of spermatozoa with a defect to the tail	19,521	12.09	7.70	0.00	100.00

^a^Trait is measured subjectively by a trained laboratory technician

The American Angus Association provided pedigree information for 1819 bulls and SNP data for 1163 bulls. The pedigree contained 6521 sires and 17,136 dams. The maximum number of SNP per bull was 54,609. Data were edited to remove SNP with a call rate of < 0.90 (*n* = 3921) and minor allele frequency of < 0.05 (*n* = 13,478). Animals with a call rate of < 0.90 were removed (n = 3). Imputation was performed by genotyping providers to the American Angus Association, so that all genotyped bulls had 54,609 SNP. After quality control filtering, a total of 38,515 SNP from 1160 animals were available for analysis. The American Angus Association also provided the SNP positions and the reference genome used by the American Angus Association was UMD3.1.

#### Statistical analysis

Beef bull fertility is known to be affected by a variety of environmental factors. A detailed model selection procedure was utilized to select the fixed effects and covariates to include in the analysis and calculate the variance components as described by [26]. In brief, we used a forward selection procedure to identify factors that significantly affected beef bull semen attributes. The factors included in the final model included location where the bull was collected (Location), the class effect of day within year (DayYear), a covariate for age of the bull on the day of collection in days (Age), a covariate for days since previous collection (DaysSince), and a covariate effect of cumulative comprehensive climate index (cumCCI) over the spermatogenesis cycle. The mathematical model used for analysis was as follows:

$${y}_{ijk}={S}_j+{W}_{pe}+ Location+ DayYear+ Age+ DaysSince+ Cum\ CCI+\varepsilon$$

where y is the phenotype being evaluated, S_j_ is a random animal effect, W_pe_ is the permanent environment effect to account for repeated measures, and ε is the residual.

The mixed model used in the current study is:

$$\left[\begin{array}{ccc}\boldsymbol{X}^{\prime}\boldsymbol{X}& \boldsymbol{X}^{\prime }{\boldsymbol{Z}}_{\boldsymbol{a}}& \boldsymbol{X}^{\prime }{\boldsymbol{W}}_{\boldsymbol{pe}}\\ {}\boldsymbol{X}^{\prime }{\boldsymbol{Z}}_{\boldsymbol{a}}& {\boldsymbol{Z}}_{\boldsymbol{a}}^{\prime }{\boldsymbol{Z}}_{\boldsymbol{a}}+\boldsymbol{\lambda} {\boldsymbol{H}}^{-\mathbf{1}}& {\boldsymbol{Z}}_{\boldsymbol{a}}^{\prime }{\boldsymbol{W}}_{\boldsymbol{pe}}\\ {}\boldsymbol{X}^{\prime }{\boldsymbol{W}}_{\boldsymbol{pe}}& {\boldsymbol{Z}}_{\boldsymbol{a}}^{\prime }{\boldsymbol{W}}_{\boldsymbol{pe}}& {\boldsymbol{W}}_{\boldsymbol{pe}}^{\prime }{\boldsymbol{W}}_{\boldsymbol{pe}}+\boldsymbol{I}{\boldsymbol{\alpha}}_{\boldsymbol{pe}}\end{array}\right]\left[\begin{array}{c}\hat{\boldsymbol{b}}\\ {}\hat{\boldsymbol{a}}\\ {}\hat{\boldsymbol{pe}}\end{array}\right]=\left[\begin{array}{c}\boldsymbol{X}^{\prime}\boldsymbol{y}\\ {}\boldsymbol{Z}^{\prime}\boldsymbol{y}\\ {}\boldsymbol{W}^{\prime}\boldsymbol{y}\end{array}\right]$$

where X is an incidence matrix relating the phenotypic observations to the fixed effects in the model (detailed above), Z_a_ is an incidence matrix relating the phenotypic observations to the additive direct genetic effects, W_pe_ is an incidence matrix relating the phenotypic observations to the additive permanent environment genetic effects, λ is the ratio of residual and additive direct genetic variance, I is an identity matrix, and α is the ratio of residual variance and additive permanent environment variance, $\hat{\boldsymbol{b}}$ is a vector of the fixed effect solutions, $\hat{\boldsymbol{a}}$ is a vector of additive direct genetic effects, $\hat{\boldsymbol{pe}}$ is a vector of additive permanent environment genetic effects, and y is a vector of phenotypic observations. Estimates for specific variance components are found in [26].

All bulls in this study had phenotypic data but because not all phenotyped animals were genotyped, and to include all available information into the analysis, both pedigree and genomic information were utilized in the genetic evaluation of beef bull fertility using a single-step genomic best linear unbiased prediction (ssGBLUP) model. In a ssGBLUP model, the numerator relationship matrix (A) is replaced with H, which augments A with the genotype information. The inverse of the H matrix is generated as follows:

$${\mathbf{H}}^{-1}={\mathbf{A}}^{-1}+\left[\begin{array}{cc}\mathbf{0}& \mathbf{0}\\ {}\mathbf{0}& {\mathbf{G}}^{-1}-{\mathbf{A}}_{22}^{-1}\end{array}\right]$$

where A^−1^ is the inverse of the pedigree-based numerator relationship matrix, A^− 1^_22_ is a subset of the numerator relationship matrix for the genotyped individuals, and G is the genomic relationship matrix for the genotyped individuals [27].

The following algorithm was used to back solve for the SNP effects for the ssGWAS [28]:

$$\boldsymbol{a}=\boldsymbol{Zu}$$

where **a** is a vector of breeding values for the genotyped individuals generated from BLUPF90, **Z** is a matrix relating individuals to phenotypes, and **u** is a vector of SNP marker effects.

The variance for the genotyped animal effects is as follows [28]:

$$\mathit{\operatorname{var}}\left(\boldsymbol{a}\right)=\mathit{\operatorname{var}}\left(\boldsymbol{Zu}\right)=\boldsymbol{G}{\sigma}_a^2=\boldsymbol{ZIZ}^{\prime}\lambda$$

where **G** is the genomic relationship matrix, **I** is an identity matrix, and **λ** is the ratio of the SNP marker effect variance and the breeding value variance.

The SNP effects were predicted utilizing the following equation:

$$\hat{\boldsymbol{u}}=\boldsymbol{IZ}^{\prime }{\left(\boldsymbol{ZI}{\boldsymbol{Z}}^{\prime}\right)}^{-1}\hat{\boldsymbol{a}}$$

where **u** is a vector of SNP marker effects, **I** is an identity matrix, **Z** is a matrix relating individuals to phenotypes, and **a** is a vector of breeding values for the genotyped individuals. Each SNP was assumed to have an equal allele substitution effect variance, and it was assumed the SNP affects followed the infinitesimal model. Thus, an unweighted ssGBLUP was performed.

SNP effects were obtained from one trivariate and six bivariate analyses. VOL, CONC, and NSP were included in a trivariate analysis. Due to convergence issues, bivariate analyses were performed among the remaining groups of traits. Initial motility, PTMot, and 3HRPTMot SNP effects were generated from three different bivariate analyses, and %NORM, PRIM, and SEC from an additional three bivariate analyses. For traits where a bivariate analysis was performed, significant SNP from both bivariate analyses are reported. Utilizing multi-trait analyses allows for multiple, genetically correlated traits to be included in the model and improves the predicative capability for each trait. The *P*-values associated with the SNP effects were obtained from the POSTGSF90 program within the BLUPF90 software suite as detailed in [29]. The *p*-value for the SNP effect is obtained by [30]:

$${p}_i=2\left(1-\Phi \left(\left|\frac{\alpha_i}{sd\left({a}_i\right)}\right|\right)\right)$$

where *α*_*i*_ is the estimate of the marker effects, sd is the standard deviation, and Φ is the cumulative standard normal function.

The *P*-values are generated by back solving for SNP effects from the breeding value estimates. This approach is possible because the fitting of animal as a random effect to generate breeding value estimates is an equivalent model to fitting all SNPs as random effects and solving for these effects directly [31].

After obtaining the SNP effects ($\widehat{\boldsymbol u}$) and their *P*-values, Manhattan plots for all nine traits were generated utilizing the CMplot package in R [32]. Utilizing the Qvalue R package [33] the -log_10_ *P*-values were converted to *p*-values so that the false discovery rate (FDR) could be calculated for each *P*-value. While there were no significant SNP identified at an FDR threshold of < 0.0001, < 0.001, < 0.01, < 0.025, or < 0.05, several significant SNP existed with a -log_10_ *P*-value threshold of 4.0 (Table 2). This less stringent threshold was chosen to allow investigation of the potential biological significance of the QTL regions identified, and is not detrimental to efforts to utilize these results in an unweighted ssGBLUP that does not specifically fit the effect of any single SNP for genomic prediction.

**Table 2:** Genomic regions identified by genome-wide association study contributing significantly to beef bull semen attributes. Significant SNP with a p-value of < 0.00001

Trait	SNP Name	-log_**10**_ ***p***-value	rsID	Chromosome	Position
**Volume**	BTB-01549373	4.01	No rsID	2	81,679,350
	Hapmap48133-BTA-96243	4.21	rs41666488	3	61,947,686
	ARS-BFGL-NGS-71827	4.26	rs109736826	3	112,997,892
	ARS-BFGL-NGS-25127	4.19	rs109268478	6	102,859,342
	Hapmap49899-BTA-18490	4.44	rs41575945	27	3,495,048
**Concentration**	BTB-01963898	4.03	rs43067163	1	133,936,071
	BTA-122016-no-rs	4.49	rs41623602	3	38,811,314
	Hapmap55203-rs29023737	4.12	rs29023737	5	3,645,270
**Number of Spermatozoa**	BTA-110980-no-rs	4.83	rs41618035	1	31,173,269
	ARS-BFGL-NGS-101891	4.28	rs109740774	6	75,137,290
	Hapmap33368-BTA-146079	4.25	rs43567728	8	89,743,053
	Hapmap44146-BTA-83959	4.07	rs41661101	9	50,922,485
	ARS-BFGL-NGS-112914	4.71	rs110005257	11	99,686,154
	ARS-BFGL-NGS-101386	4.55	rs110190516	17	67,017,094
	ARS-BFGL-NGS-85970	4.04	rs108993490	24	16,118,203
**Initial Motility**	Hapmap48211-BTA-120784	4.69	rs41623436	1	152,412,536
	ARS-BFGL-NGS-74920	4.54	rs109798673	2	827,626
	BTB-00319289	4.13	rs43526428	7	73,777,772
	Hapmap59148-ss46527122	4.10	rs29003479	11	63,430,172
	BTB-01751684	4.04	rs42861585	23	46,295,105
	Hapmap23061-BTC-074055	4.05	rs109512383	25	30,964,321
**Post-thaw Motility**	Hapmap48211-BTA-120784	4.75	rs41623436	1	152,412,536
	ARS-BFGL-NGS-74920	5.63	rs109798673	2	827,626
	Hapmap45703-BTA-105835	4.10	rs41611445	2	82,083,660
	BTB-01323835	4.22	rs42446055	4	89,708,810
	BTB-01537954	4.32	rs42653645	11	25,036,830
	ARS-BFGL-NGS-41002	4.52	rs110418161	16	36,415,574
	ARS-BFGL-NGS-111058	4.82	rs109719529	17	56,344,620
**Three Hour Post-thaw Motility**	Hapmap59148-ss46527122	4.50	rs29003479	11	63,430,172
	ARS-BFGL-NGS-111058	4.49	rs109719529	17	56,344,620
**Percentage of Normal Spermatozoa**	BTA-50285-no-rs	4.41	rs41606310	1	7,669,386
	ARS-BFGL-NGS-43775	5.94	rs110964837	2	73,209,337
	BTA-91078-no-rs	4.85	rs41594758	3	43,031,873
	ARS-BFGL-NGS-25359	4.71	rs109928164	5	70,049,126
	BTA-74480-no-rs	4.66	rs41591913	5	84,578,337
	BTA-98744-no-rs	4.68	rs41666416	10	68,089,016
**Primary Abnormalities**	BTA-115758-no-rs	4.11	rs41566683	1	36,903,496
	Hapmap57078-ss46526391	4.95	rs41255529	1	60,956,897
	ARS-BFGL-NGS-44433	4.22	rs110487590	1	63,799,085
	BTB-01831345	4.51	rs42940192	4	10,648,384
	ARS-BFGL-NGS-80666	4.05	rs110425782	20	68,324,872
	Hapmap42996-BTA-60916	4.16	rs41646489	22	33,690,494
**Secondary Abnormalities**	Hapmap42996-BTA-60916	4.28	rs41646489	22	33,690,494

### QTL analysis

To account for linkage disequilibrium in the *Bos taurus* genome, QTL regions were formed 250,000 kilobases upstream and downstream from the significant SNP locations [34]. The regions were utilized to identify previously reported QTL which were near the significant SNP (±250,000 kilobases). The QTL regions were identified utilizing the cattle QTL database [35]. For each trait, previously reported QTL were identified close to significant SNP. Where possible, SNP were identified by the corresponding rs number. If no rs number was available, the SNP name was utilized. In addition, the same QTL region boundaries were used to identify genes near the significant SNP using the National Center of Biotechnology Information (NCBI) database. Putative candidate genes were identified near significant SNP and examined for biological meaning. The genes specifically associated with beef bull semen attributes were identified based on the gene functions [36]. The gene list was then used to perform a functional annotation analysis utilizing the Database for Annotation, Visualization, and Integrated Discovery (DAVID 6.8) [37, 38] for each individual beef bull semen attribute.

## Results and discussion

**Fig. 1:**
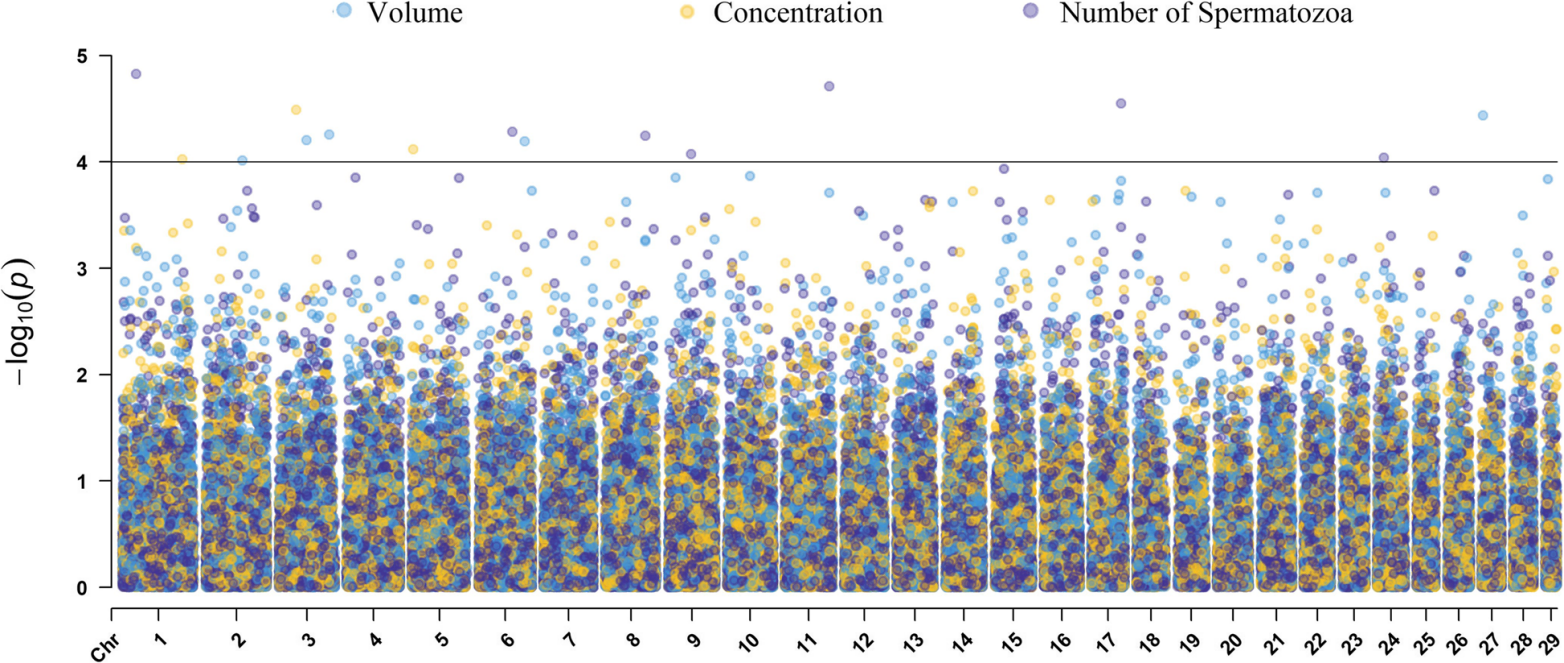
Manhattan plot showing the result of genome-wide association mapping for volume, concentration, and number of spermatozoa with a significance threshold of 4.0. A trivariate analysis was performed

**Fig. 2:**
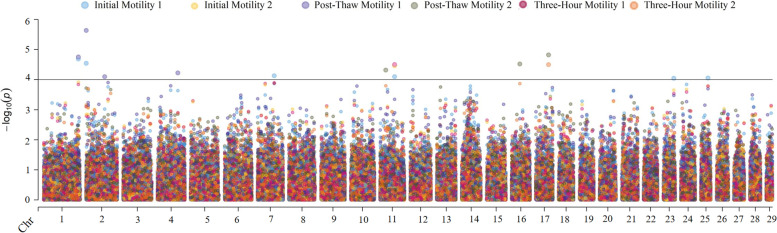
Manhattan plot showing the result of genome-wide association mapping for motility traits with a significance threshold of 4.0. A bivariate analysis was performed, thus significant SNP from both bivariate analyses are reported

**Fig. 3:**
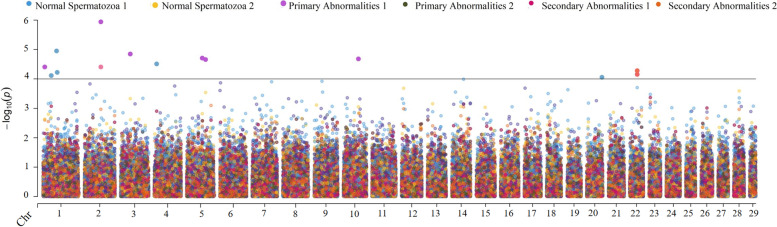
Manhattan plot showing the result of genome-wide association mapping for percentage of normal spermatozoa and abnormality traits with a significance threshold of 4.0. A bivariate analysis was performed, thus significant SNP from both bivariate analyses are reported

Significant SNP were identified for all beef bull semen attributes. Significant SNP are reported in Table 2 and Manhattan plots are provided in Figs. 1, 2, and 3. For each trait, previously reported QTL were identified close to significant SNP (Table 3). Putative candidate genes specifically associated with beef bull semen attributes are outlined in Table 4.

**Table 3:** Previously reported fertility-associated QTL regions which overlap significant SNP identified for beef bull semen attributes in this study

Trait	SNP Name	rsID	Chr:Position	QTL Trait	Source
Volume	BTB-01549373	No rsID	2: 81679350	Conception rate	[39]
				Non-return rate	[9]
	Hapmap49899-BTA-18490	rs41575945	27:3495048	Non-return rate	[10]
Number of Spermatozoa	BTA-110980-no-rs	rs41618035	1:31173269	Conception rate	[40]
				Inseminations per conception	[11]
				Non-return rate	[11]
	ARS-BFGL-NGS-101891	rs109740774	6:75137290	First service conception	[39]
	Hapmap33368-BTA-146079	rs43567728	8: 89743053	Conception rate	[39]
	Hapmap44146-BTA-83959	rs41661101	9:50922485	Non-return rate	[41]
				Structural soundness of feet, legs, penis, and prepuce	[42]
	ARS-BFGL-NGS-101386	rs110190516	17: 67017094	Non-return rate	[43]
	ARS-BFGL-NGS-85970	rs108993490	24:16118203	First service conception rate	[39]
				Conception rate	[39]
Initial Motility	ARS-BFGL-NGS-74920	rs109798673	2: 827626	Conception rate	[39]
				Fertilization rate	[38]
	BTB-00319289	rs43526428	7:73777772	Conception rate	[39, 44]
				Daughter pregnancy rate	[21, 44]
				Sire conception rate	[45]
				Sexual precocity	[45]
	Hapmap59148-ss46527122	rs29003479	11:63430172	Scrotal circumference	[46]
	Hapmap23061-BTC-074055	rs109512383	25: 30964321	Conception rate	[39]
				Scrotal circumference	[46]
				Sperm average path velocity	[12]
Post-thaw Motility	ARS-BFGL-NGS-74920	rs109798673	2:827626	Conception Rate	[39]
				Fertilization Rate	[38]
	Hapmap45703-BTA-105835	rs41611445	2: 82083660	Conception rate	[39]
				Non-return rate	[9]
	BTB-01537954	rs42653645	11:25036830	Sperm motility	[12, 16]
Three-hour Post-thaw Motility	Hapmap59148-ss46527122	rs29003479	11: 63430172	Scrotal circumference	[46]
Percentage of Normal Spermatozoa	BTA-50285-no-rs	rs41606310	1: 7669386	Daughter pregnancy rate	[22]
	ARS-BFGL-NGS-43775	rs110964837	2:73209337	Conception rate	[12, 17, 39]
				Non-return rate	[9]
	ARS-BFGL-NGS-25359	rs109928164	5: 70049126	Daughter pregnancy rate	[17]
	BTA-74480-no-rs	rs41591913	5:84578337	Conception Rate	[17]
	BTA-98744-no-rs	rs41666416	10: 68089016	Fertility index	[23]
				Scrotal circumference	[47]
Primary Abnormalities	BTA-115758-no-rs	rs41566683	1: 36903496	Conception rate	[39, 40]
	Hapmap57078-ss46526391	rs41255529	1:60956897	Conception rate	[39, 40]
				First service conception	[39]
				Non-return rate	[9]
				Scrotal circumference	[46]
	ARS-BFGL-NGS-44433	rs110487590	1: 63799085	Conception rate	[17, 40]
				Daughter pregnancy rate	[17]
				Non-return rate	[9]
				Scrotal circumference	[46]
				Testicular hypoplasia	[48]
	BTB-01831345	rs42940192	4:10648384	Non-return rate	[49, 50]
				Male fertility	[51]

**Table 4:** Significant SNP with putative candidate genes for semen production traits

SNP	Trait	Chromosome: Pos	Closest gene	Biological Process	Distance from gene (bp)	Relative Location of SNP to gene
**rs109798673**	Initial Motility	2:827626	HERC2	Causes reduced spermatozoa motility	0	Within
	Initial Motility	2:827626	OCA2	Contributes to spermatid development	196,400	Downstream
	Post-thaw Motility	2:827626	HERC2	Caused reduced spermatozoa motility	0	Within
	Post-thaw Motility	2:827626	OCA2	Contributes to spermatid development	196,400	Downstream
**rs110425782**	Primary Abnormalities	20:68324872	LOC101902976	Necessity for sperm motility	108,733	Downstream

For VOL, five strongly associated SNP were identified, including BTB-01549373, rs41666488, rs109736826, rs109268478, and rs41575945 (Table 2). Three QTL regions associated with conception rate or non-return rate in dairy cattle [10, 39, 52] were near BTB-01549373 and rs41575945 (Table 3). While conception and non-return rate are generally associated with female fertility, it is interesting that SNP significantly associated with male fertility traits are also near these QTL regions. This is evidence that the ability to successfully reproduce is dependent not only on the female, but also on the semen quantity and quality of the bull [52]. This may also indicate that although the few reported genetic correlations between male and female fertility are low [47, 53, 54], there may be some pleiotropy whereby mutations control aspects of both male and female fertility.

Three significant SNP were identified for CONC, which included rs43067163, rs41623602, and rs29023737 (Table 2). A previously reported mature height QTL region was close to rs41623602 [46]. In addition, a previously reported QTL region associated with weaning weight and mature height was in proximity to rs41623602 (Table 3) [46]. While there are few studies that directly relate size of the bull to the semen quality, weight and height are generally associated with maturity, and the association could be indicative that more mature, larger bulls have greater semen production. In the current study, weight and hip height of the bull at the time of collection did not contribute significantly to concentration [26].

Seven SNP had significant associations with NSP (Table 2). Three different previously reported conception rate QTL regions [39, 40] neighbored significant SNP for NSP. In addition, previously reported QTL regions associated with non-return rate were in the vicinity of two significant SNP from the current study [11, 41]. Like conception rate, non-return rate is usually related to female fertility, but this association provides further validation that male fertility traits are integral in the reproductive success of the herd [52] and/or that male and female fertility share a genetic correlation due to pleiotropy in these regions of the genome.

For IMot, 6 SNP on 6 different chromosomes were significant, including rs41623436, rs109798673, rs43526428, rs29003479, rs42861585, and rs109512383. One SNP, rs29003479, on chromosome 11 was significant in both bivariate analyses for initial motility and the other five were only significant in one of the analyses. The SNP significant in both bivariate analyses was near a previously reported scrotal circumference QTL region [46]. Scrotal circumference has been reported to be strongly and positively genetically correlated with motility [6, 55]. A previously reported QTL for sperm average path velocity overlapped the QTL region for rs109512383 [12]. Sperm average path velocity is a measure obtained from a computer-assisted semen analysis and represents the average trajectory of the sperm cell. The trajectory of the spermatozoa is dependent upon the ability of the spermatozoa to move, thus providing further evidence of the relationship between initial motility and sperm average path velocity. A QTL region for sire conception rate [45] was in the vicinity of rs43526428, a SNP significant for IMot (Table 3). In addition, a reported conception rate QTL region [39, 56], and in the current study, a significant SNP for IMot was close to this region. Once the spermatozoa are deposited into the female reproductive tract, it is essential for the sperm cells to progress towards the oocyte for fertilization and, therefore conception [44]. Interestingly, a QTL region found to influence sexual precocity [45] is near a significant SNP associated with IMot in this study. This is interesting because the average age of bulls in this study was just over two years old, with the youngest bull in the evaluation just under a year old. Bull studs often collect very young bulls and producers demand the semen because these bulls often have more desirable EPD (albeit unproven and lower accuracy) than their older stud mates. Young bulls must have the libido and semen quality to produce viable sperm, so the relationship between sexual precocity and initial motility could indicate young bulls are able to produce a viable ejaculate. Quantitative trait loci associated with milk composition [13, 14] and interval to first estrus after calving [15] were found neighboring rs41623436. A recent study [25] found the same results with crossbred beef bulls, where a significant SNP window was within a QTL for interval to first estrus after calving. Return to estrus after calving is dependent on the hormones gonadotropin releasing hormone (GnRH), luteinizing hormone (LH), and follicle stimulating hormone (FSH). The production of spermatozoa is also dependent on GnRH, signaling the release of LH to induce the Leydig cells to produce testosterone. Testosterone paired with FSH signaling to the Sertoli cells causes the production of male gametes. It could be speculated that females which return to estrus more quickly after calving have increased hormone signaling, which could indicate males which have significant SNP near these QTL additionally have stronger hormone signaling; and therefore; produce more and higher quality gametes.

Seven SNP were identified for PTMot (Table 3). A QTL region on chromosome 11 which reportedly influences spermatozoa motility [12, 16] encompassed rs42653645, a SNP significant for PTMot. Two different conception rate QTL regions have been reported [39] near two SNP significant for PTMot in this study. As motile spermatozoa are necessary for conception, this is a logical relationship.

Six significant SNP were identified for %NORM (Table 2). The SNP were rs41606310 on chromosome one, rs110964837 on chromosome two, rs41594758 on chromosome three, rs109928164 on chromosome five, rs41591913 on chromosome five, and rs41666416 on chromosome ten. Notably, rs110964837 was significant in both bivariate models where percentage of normal spermatozoa was evaluated with either PRIM or SEC. The SNP rs110964837 was one of the two SNP near conception-rate QTL regions and a non-return rate QTL region [9, 12, 17, 39]. Previously reported scrotal circumference QTL regions [46], and a significant SNP for %NORM in the current study was near the reported region. Two conception-rate QTL regions and a non-return rate QTL region [9, 12, 17, 39] were close to SNP significantly associated with %NORM. As previously discussed, SNP significantly associated with male fertility traits nearby fertility-related QTL regions is further evidence that the quality of a bull’s semen is important for conception or that the same genes underlie attributes affecting conception in both males and females.

Primary abnormalities had six strongly associated SNP: rs41566683, rs41255529, rs110487590, rs42940192, rs110425782, and rs41646489 (Table 2). A previously reported scrotal circumference QTL region found in a population of Angus bulls [46] was near rs41255529, a SNP significant for PRIM. Previously reported genetic correlations between scrotal circumference and PRIM indicate increased scrotal circumference results in fewer PRIM [41, 44]. Three significant SNP for PRIM were in proximity to QTL regions associated with conception rate and non-return rate [39, 40, 46]. Primary abnormalities are abnormalities of the head, and without proper head formation, the sperm cell cannot penetrate the zona pellucida on the oocyte and cause conception [44].

All genes near SNP significant for IMot are outlined in Table 4. A SNP contributing significantly to IMot was within the gene E3 ubiquitin protein ligase 2 (HERC2). In addition, the significant SNP within HERC2 was also downstream of the gene melanosomal transmembrane protein (OCA2). The gene OCA2 is most commonly associated with albinism; however, it also contributes to spermatid development [36]. Reported in Table 4 are additional genes identified within the QTL regions for IMot.

A SNP significant for PTMot was in proximity to collagen like tail subunit of asymmetric acetylcholinesterase (COLQ), which contributes to the structural integrity of the extracellular matrix and heparin binding [57]. It is important to note heparin is an important enhancer of capacitation of the bovine spermatozoa [19]. Furthermore, heparin-binding proteins allow the spermatozoa to undergo the acrosome reaction [20]. In addition, a significant SNP for PTMot was in proximity to the heat shock protein family B (small) member 8 (HSPB8) protein. Elevated heat shock protein levels have been speculated to be associated with immature spermatozoa [58]. This research addressed human infertility and found that an increase in the 70-kDa heat shock protein HspA2 resulted in spermatozoa not completing the phases of changing the plasma membrane during epididymal maturation and having larger amounts of cytoplasmic proteins in mature spermatozoa [58]. As previously discussed, heat shock protein can affect the maturation of the spermatozoa and cause infertility [58]. Reported in Table 4 are additional genes identified near SNP affecting PTMot.

Although not within ±250,000 kilobases of the significant SNP, a pertinent gene nearby was the major facilitator superfamily domain containing 14A (MFSD14A) gene. Also known as HIAT1, it was 300,000 kilobases upstream of the SNP. The MFSD14A gene is important for spermatid nucleus differentiation and sperm mitochondrion organization. It has been reported that mice with a disruption to the MFSD14A gene are infertile due to incomplete acrosome formation and round head defects [59].

Primary abnormalities had a significant SNP in the vicinity of a gene on chromosome 20 (Table 4). Primary abnormalities had a significant SNP within sperm mitochondrial-associated cysteine-rich protein-like (LOC101902976). Sperm-mitochondrial cysteine-rich protein is located in the mitochondrial capsule and plays a role in sperm motility [42]. While the mitochondria are located in the midpiece of the spermatozoa rather than the head, it is still important to note it is associated with male fertility.

## Conclusion

Several QTL associated with fertility traits were identified in this study and validated by previously published literature. In addition, genes within the QTL regions for beef bull semen attributes were discussed. While a few gene functions could be associated with beef bull semen attributes, some of the genes found in the current study do not have any known association with fertility traits reported in previous literature and have unknown related physiological association. Results from the current study, paired with findings in previous literature, validate that bull fertility traits are controlled by genetic factors. Identification of the QTL regions and SNP positions associated with these traits provides the knowledge necessary to enrich these regions in future iterations of SNP chips used within the cattle industries, and thus increase genomic prediction accuracies for these traits should the industry add them to routine genetic evaluation. The increased use of genomic testing in tandem with the number of phenotypes routinely recorded by bull semen collection facilities makes identification of beef bulls with superior genetic merit for fertility traits feasible. This advancement would increase profitability of beef producers by improving fertilization rate, increasing overall herd reproductive rate, and providing producers another genetic tool to use in making selection decisions.

## Data Availability

The data that support the findings of this study are available from the American Angus Association and corresponding AI collection centers but restrictions apply to the availability of these data, which were used under license for the current study, and so are not publicly available. Data are available from the original data providers and/or the authors upon reasonable request and only with written permission of the original data providers and only in cases where this is consistent with the rules set forth in the data transfer agreement signed by both parties.
